# Barriers and drivers to consuming neglected and underutilized species: evidence from six European countries

**DOI:** 10.1038/s41598-025-24443-4

**Published:** 2025-11-19

**Authors:** Simoun Bayudan, Lukas Zagata, Lucie Plzakova, Hans De Steur, Joachim Jietse Schouteten

**Affiliations:** 1 Department of Agricultural Economics, Faculty of Bioscience Engineering, Ghent University, Ghent, 9000 Belgium; 2 Department of Humanities, Faculty of Economics and Management, Czech University of Life Sciences Prague, Prague, 16521 Czech Republic

**Keywords:** Diversity, Multicultural, Ancient crops, Barriers and drivers, Motivation, Consumer behavior, Psychology and behaviour, Socioeconomic scenarios

## Abstract

Neglected and underutilized species (NUS) are considered important for agrobiodiversity and landscape health, yet there is limited evidence on factors that encourage NUS consumption. Hence, this study examined drivers and barriers among consumers in including NUS products in their diets, following the premises of innovation resistance and food choice. An online consumer survey was conducted in six European countries to measure consumers’ perceived barriers, drivers, and willingness to consume NUS. A total of 4802 responses from participants (balanced in age and gender) were analyzed (Mean age: 43.2 ± 13.0 years, 50.6% female, 49.4% male). Furthermore, hierarchical multiple linear regression showed that respondents had a neutral outlook towards consuming NUS. Psychological barriers seemingly had similar negative influences on intentions to consume NUS together with functional barriers. Motives to eat food alongside concerns for the environment and health and taste properties of food positively influenced NUS consumption intentions. Put together, understanding the balance of both driving and hindering factors for NUS consumption can therefore promote NUS consumption across broader audiences.

**Supplementary Information:**

The online version contains supplementary material available at 10.1038/s41598-025-24443-4.

## Introduction

### The present context of agrobiodiversity

 One of the means to which agrobiodiversity can be preserved is through the promotion of neglected and underutilized species (NUS)^1,2^. NUS, also known as minor, orphan, and indigenous crops, are traditionally grown species that are not mass-produced relative to mainstream crops such as rice, wheat, and maize^3,4^. Some NUS examples include lupins and triticale^5,6^; yet these are produced mostly for feed or for non-human consumption^7^. While other types of more niche crops have been cultivated in the past for human consumption, such as oat, millet, and buckwheat, the distribution of how diverse crops are produced around the globe remains inconsistent and this has implications on what is available to eat^8^.

The prevailing knowledge about the cultivation of NUS points to a wide array of benefits for the food chain. Some NUS have been deemed to enhance soil quality by conserving or regenerating lost nutrients^4^. Additionally, the sustained cultivation of NUS has been linked to better food security outcomes^9–12^. Several innovative food products can also be derived from these crops such as plant-based beverages from oats, pasta, and bakery products from buckwheat and millets^13–15^.

From a consumption perspective, having more NUS sources seems to be advantageous for the diet. First, consuming NUS may improve an individual’s dietary diversity level and health outcomes^16^. Second, many NUS are rich in various micro- and macronutrients that help maintain physiological processes^5,17,18^. Third, NUS can act as alternative sources of proteins, fiber, and even vitamins^19^.

Despite the potential utility of NUS, the consumption of these crops and commodities remains dismal at best, and that biodiversity is not as incorporated in mainstream diets^20^. A review conducted mentions that the lack of a stable market for these crops and competition against mainstream produce might hinder crop diversification^21^. Furthermore, there is a paucity of studies regarding consumer perspectives on NUS. While some products made with NUS already exist in the market, there remains much room for new products that consumers will be willing to adopt^22,23^.

Hence, this study aims to establish drivers and barriers prevailing among European consumers related to the consumption of NUS. First, this study will determine which types of barriers discourage consumption for NUS, following the Innovation Resistance Theory^24^, since similar to prior studies, consumers tend to be amenable towards innovations related to traditional food products^25^. Alongside this, other psychological attributes will be examined to identify what types of factors can drive NUS consumption, as well as the extent to which barriers affect these drivers. Finally, socio-demographic profiling of individuals with varying extents of barriers perceived to consumption will be done.

### Conceptual framework and hypotheses

#### The innovation resistance theory

Consumer resistance to innovations is theorized to be driven by two main factors – psychological and functional barriers. The use of this theory has been applied in various studies and in various sectors, with innovation resistance being scoped among consumers of mobile food delivery applications, eco-friendly cosmetic products, and organic food commodities^26–29^.

Functional barriers are related to the consumer’s physical experiences when dealing with a certain innovation^24^. The theory operationalizes functional barriers by considering the utility and overall value of a given innovation. In applying this to food consumption, several determinants which are functional in nature arise in the decision-making process of the consumer for their food choices^30^. For instance, the health and food safety aspects of NUS as well as the effort needed to prepare eating these commodities can impact an individual’s propensity to follow better diets, and consequently, they may be functional barriers to eating^31^. Therefore, this study sets the following hypothesis concerning the consumption of NUS:

H1a. The increased perception of functional barriers such as those related to health, safety, and effort in eating NUS diminishes consumers’ intentions to consume products made with NUS.

Conversely, psychological barriers are concerned with conceptualizations that individuals associate with certain food choices, according to the Innovation Resistance Theory. These psychological barriers are driven by intrapersonal factors such as an individual’s preconceived notions about an object in question^30^. Furthermore, the impacts of these psychological barriers are relevant in food consumption^32^. For example, it has been shown that individuals avoid food with an unfamiliar image such as unusual food due in part to hesitation about modifying dietary habits^33^. Therefore, this study hypothesizes that:

H1b. The increased perception of psychological barriers linked to the unusual image of food and a consumer’s hesitation to modify dietary habits related to NUS diminishes consumers’ intentions to consume products made with NUS.

#### Personal attributes driving food consumption

Innovation resistance and consumer intentions appear to have interplays with environmental concerns, existing buying behaviors regarding unfamiliar food, and even health issues^28,29^. Therefore, in this study, an individual’s motivations to eat new food, attitudes towards health and taste, as well as environmental beliefs were identified as potential drivers towards the consumption of NUS. The following section then describes each of these factors, as well as how their hypothesized associations with consuming products made with NUS were constructed.

In operationalizing the concept of motivations to eat new food, the MENF scale was used. The Motivations to Eat Novel Food (MENF) scale was developed with the overall goal of providing a more concise measurement of particular motives accorded by consumers towards unfamiliar and new products^34^. Building upon the literature on food neophobia and disgust, the MENF scale considers simultaneously both approach and avoidance motives that an individual possesses. However, in the case of NUS products, the term ‘novel’ may not necessarily apply, as this designation is contingent on food regulations, at least in the EU region (Regulation 2015/2283). Here, the focus of the study lies then on attributing NUS products as relatively ‘new’ in the sense that they may not have been largely consumed by consumers in the past as they have been unconventional goods. Thus, when faced with contemporary and ‘new’ products, there appears to be an equally relevant risk of consumers rejecting these products out of fear or aversion to try^34^ or otherwise. Hence, the following is hypothesized:

H2a. Consumers with stronger motivations to eat new food likewise have increased intentions to consume products made with NUS.

Aside from consumer motives towards new food, the widely used Health and Taste Attitudes Scale (HTAS) measures consumer attitudes related markedly to various intrinsic properties of food products^35^. As NUS are often cited for their potential health benefits, there might be trade-offs among the decision-making processes of consumers when they consider other product qualities of NUS such as taste. From this, the following is forwarded:

H2b. Consumers with stronger attitudes towards the general health and taste qualities of food have increased intentions to consume products made with NUS.

Going beyond food-related attitudes, another plausible variable affecting consumption is the belief of consumers towards environmental issues. Prior studies have shown that environmental beliefs seem to be associated with higher intentions to pursue more sustainable food consumption behaviors^36^, yet there still exists a gap between sustainability-oriented intentions and actual behaviors^37^. Given that NUS are also forwarded as potential protective elements of the landscape^4^, it is worthwhile to investigate the dynamics of personal environmental beliefs towards NUS commodities. Therefore, this study hypothesizes that:

H2c. Consumers with stronger personal beliefs regarding environmental issues have increased intentions to consume products made with NUS.

Linking the above-discussed drivers with functional and psychological barriers, this study will also explore whether perceived barriers can moderate an individual’s motives to eat NUS products. Therefore:

H3. Consumers’ perceived barriers will negatively reduce the effect of motivation to eat new food, health and taste attitudes, and environmental beliefs towards intentions to consume products made with NUS.

Finally, socio-demographic characteristics and country-level differences were taken into consideration regarding the relationships on barriers and drivers towards consumption intentions for NUS. Socio-demographic characteristics have been extensively studied in food consumption, particularly with certain associations among new food products^38–40^. For instance, some associations have been found among gender, wealth, education status and dietary lifestyles^41,42^. The following is thus hypothesized:

H4. Significant differences exist among consumers with distinct socio-demographic characteristics, including their place of residence, in relation to drivers and barriers towards products made with NUS.

**Fig. 1:**
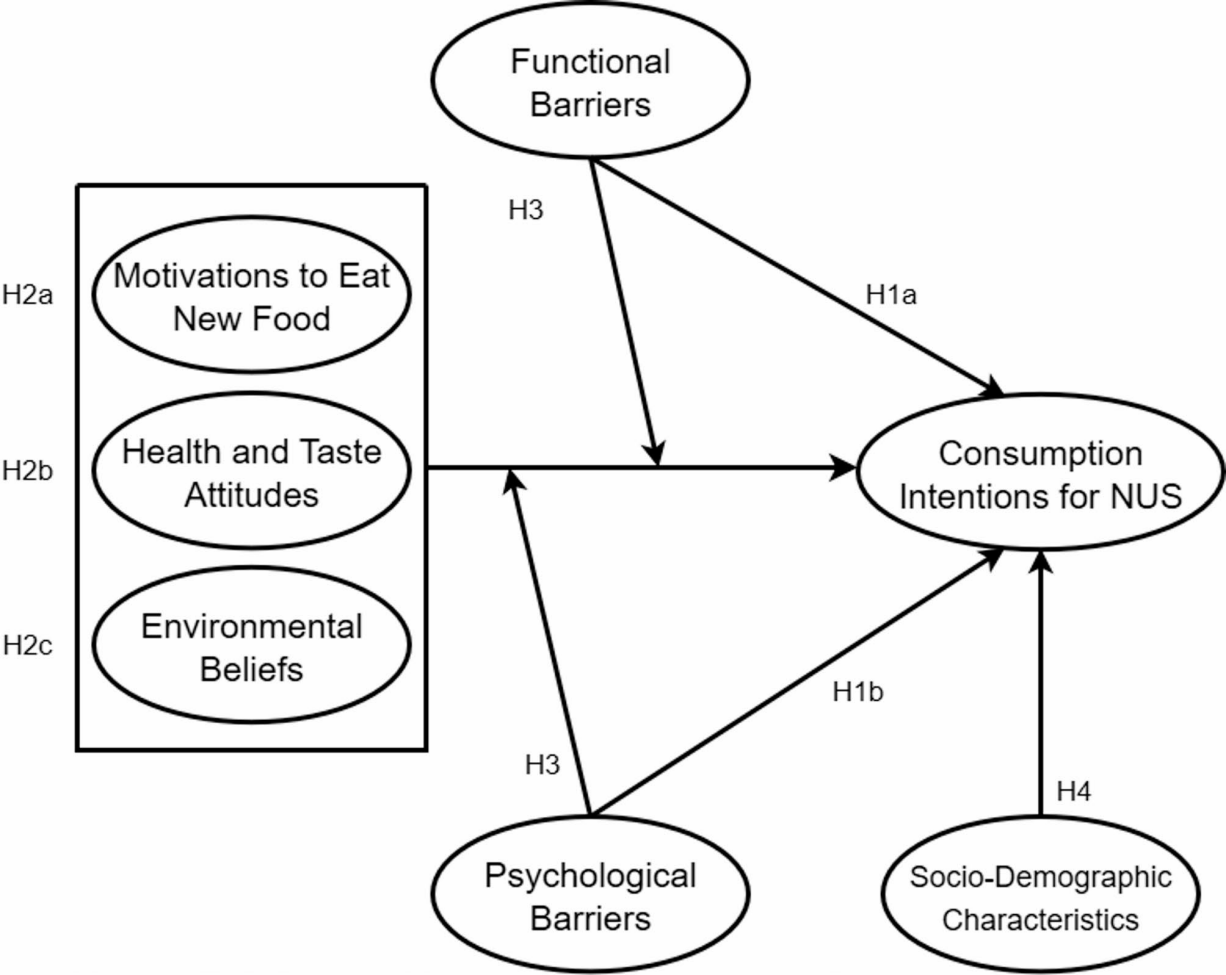
Conceptual framework of the study

Figure 1 presents a summary of the conceptual framework along with the proposed hypotheses of this study.

## Results

### Overview of perceived functional and psychological barriers towards consuming NUS

After running the exploratory factor analysis procedure, one-factor solutions were extracted for the analysis on the functional barriers and the psychological barriers respectively. Therefore, due to the one-factor solution observed each time, a summated scale was composed for each barrier type, and the reliability measures were computed for each as well (ω). The McDonald Omega reliability values for the items are adequate, indicating the agreement of the different scale items when linked to the single latent construct (either FB or PB). Table 1 provides the factor loadings of the specific statements used in the survey for each extracted factor as well as the resulting reliability values.

**Table 1:** Factor loadings for extracted components from perceived barriers

Item	FB	Item	PB
I am worried food made with NUS are not safe to eat.	0.82	Food made with NUS are not enjoyable.	0.78
I am concerned that NUS food are not nutritious enough.	0.81	I don’t want to change my eating habits or routine.	0.76
I am not sure if these products made with NUS are natural.	0.79	NUS are only eaten in poorer households.	0.74
I am not convinced of the benefits of consuming NUS food.	0.71	I think humans are meant to eat only conventional crops.	0.72
It takes too long to prepare meals using underutilized crops.	0.62	Products made with conventional crops are enough for me.	0.67
Food made with NUS are not healthier than conventional crops.	0.57	Food made from NUS seem unusual.	0.59
Scale reliability measure ω = 0.83		Scale reliability measure ω = 0.80	

While initially both psychological and functional barriers were not strongly perceived by the participants, psychological barriers (M = 2.69 ± 0.70) were perceived slightly less compared to functional barriers (M = 2.74 ± 0.68) upon comparing overall mean scores for all participants (t = 6.89, df = 4801, p-val < 0.01, d = 0.10). A closer inspection of the functional barriers (Appendix A1) further revealed nuances as to what exact barriers are perceived. Notably, while the different individual barriers were seemingly rated the same, the participants reported not being convinced of the benefits of NUS being the highest on average, possibly indicating issues on familiarity and promotion of NUS. Additionally, while safety was the lowest perceived barrier, the relatively similar scores again potentially indicate concerns on familiarity and the presentation of NUS as safe to consume, especially as there are NUS commodities that have commercial potential. Regarding the psychological barriers, participants were generally in agreement with the notion that conventional crops are already sufficient, and that products made with NUS seem unusual. Conversely, participants on average still viewed that NUS have an associated image with consumption in poorer households, and this might relate to cultural associations and uses of these food products.

### Overview of consumption intentions for NUS and food-related attitudes

Most of the respondents were generally neutral in trying out NUS products. However, they showed higher scores for approach motivations to eat new food compared to avoidance motivations. The respondents across all the countries also reflected higher scores pertaining to environmental beliefs. When it comes to the health and taste properties of food products, moderate scores were received, demonstrating that these quality considerations were not of low priority when making food choices.

Table 2 shows an overview of the significant differences associated with these variables across the countries. These consistent differences, albeit with small to medium effect sizes, indicate some degree of cross-national effects in relation consuming NUS. For example, respondents from Austria registered the lowest scores for avoidance motivations towards new food. Similarly, participants from Belgium had the lowest scores when it comes to their overall attitudes towards general health and natural properties of the food products they consume, compared to the others. Regarding the environmental beliefs variable, respondents from Spain and Italy scored higher compared to the others.

**Table 2:** Country level summary measures of barriers (FB and PB), drivers (MENF, HTAS, ENV), and consumption intentions (INT)

Variable		Overall *N* = 4802	Austria *N* = 800	Belgium *N* = 757	Czech Rep. *N* = 824	Italy *N* = 750	Serbia *N* = 889	Spain *N* = 782	Difference Test*	η²
		X̄ ± S.D.	X̄ ± S.D.	X̄ ± S.D.	X̄ ± S.D.	X̄ ± S.D.	X̄ ± S.D.	X̄ ± S.D.		
Intentions to Consume NUS	INT	3.47 ± 0.85	3.51 ± 0.91	3.35 ± 0.82	3.17 ± 0.87	3.64 ± 0.84	3.46 ± 0.73	3.69 ± 0.86	F_w(5,2220)_ = 39.1 **p-val < 0.01**	0.04
Approach Motivations	MENF-AP	3.43 ± 1.05	3.42 ± 1.09	3.47 ± 0.90	3.00 ± 1.14	3.50 ± 1.06	3.59 ± 0.89	3.59 ± 1.05	F_w(5,2221)_ = 33.0 **p-val < 0.01**	0.04
Avoidance Motivations	MENF-AV	2.1 ± 0.92	1.85 ± 0.87	2.34 ± 0.84	2.10 ± 0.92	2.21 ± 1.03	2.02 ± 0.83	2.11 ± 0.98	F_w(5,2220)_ = 28.8 **p-val < 0.01**	0.03
Health and Taste Attitudes	HTAS	3.21 ± 0.66	3.21 ± 0.74	3.06 ± 0.65	3.20 ± 0.68	3.36 ± 0.59	3.10 ± 0.61	3.37 ± 0.63	F_w(5,2227)_ = 34.6 **p-val < 0.01**	0.03
Environmental Beliefs	ENV	3.87 ± 0.90	3.81 ± 1.00	3.87 ± 0.88	3.58 ± 0.90	4.11 ± 0.85	3.79 ± 0.77	4.08 ± 0.88	F_w(5,2221)_ = 41.0 **p-val < 0.01**	0.04
Functional Barriers	FB	2.74 ± 0.68	2.59 ± 0.69	2.79 ± 0.65	2.68 ± 0.61	2.69 ± 0.79	2.84 ± 0.58	2.86 ± 0.73	F_w(5,2214)_ = 20.5 **p-val < 0.01**	0.02
Psychological Barriers	PB	2.69 ± 0.70	2.48 ± 0.73	2.70 ± 0.69	2.73 ± 0.67	2.76 ± 0.73	2.72 ± 0.63	2.75 ± 0.73	F_w(5,220)_ = 16.8 **p-val < 0.01**	0.02

Note. *Significant differences marked in bold, from a Welch-ANOVA for country comparisons

### Drivers of consumption for NUS products

Table 3 shows the significant Pearson correlation values for all variables used. Environmental beliefs, approach motivations to new food, and health and taste properties were positively associated with consumption intentions for NUS. Avoidance towards new food and both psychological and functional barriers were inversely related to consumption intentions.

**Table 3:** Mean-centered Pearson’s correlation values.

VAR	INT	ENV	MENF-AP	MENF-AV	HTAS	FB	PB
INT							
ENV	0.42						
MENF-AP	0.50	0.30					
MENF-AV	−0.25	−0.15	−0.31				
HTAS	0.33	0.21	0.18	−0.16			
FB	−0.38	−0.20	−0.22	0.44	−0.18		
PB	−0.44	−0.24	−0.33	0.50	−0.24	0.74	

Note. All correlation values were significant (p-val < 0.01)

Extending the correlation analysis, results of the hierarchical multiple regression are shown in Table 4 and significant predictors for consumption intentions of NUS were identified. Severe collinearity was not detected after examining the VIF values.

**Table 4:** Predictors to consumption intentions for NUS products

Variable	Block 1			Block 2		
	Std. β	*p*-val	VIF	Std. β	*p*-val	VIF
Age	0.04	**< 0.01**	1.48	0.04	**< 0.01**	1.49
Gender: Male^a^	−0.02	**0.04**	1.07	−0.02	**0.04**	1.07
Country: Austria^b^	−0.08	**< 0.01**	1.88	−0.08	**< 0.01**	1.90
Country: Belgium^b^	−0.11	**< 0.01**	1.78	−0.11	**< 0.01**	1.79
Country: Czechia^b^	−0.13	**< 0.01**	1.94	−0.13	**< 0.01**	1.95
Country: Italy^b^	−0.04	**0.01**	1.73	−0.04	**0.01**	1.74
Country: Serbia^b^	−0.07	**< 0.01**	1.86	−0.06	**< 0.01**	1.87
Occupation: Student^c^	−0.02	**0.05**	1.31	−0.02	0.12	1.31
Occupation: Other^c^	0.01	0.57	1.16	0.01	0.56	1.16
Education: Post-Secondary^d^	−0.02	0.16	1.12	−0.01	0.30	1.13
Area of Residence: Rural^e^	0.02	0.10	1.11	0.02	0.09	1.11
Environmental Beliefs (ENV)	0.21	**< 0.01**	1.21	0.20	**< 0.01**	1.22
Approach Motives (MENF-AP)	0.34	**< 0.01**	1.36	0.31	**< 0.01**	1.40
Avoidance Motives (MENF-AV)	0.07	**< 0.01**	1.48	0.04	**< 0.01**	1.62
HTAS Score	0.15	**< 0.01**	1.19	0.14	**< 0.01**	1.24
Functional Barriers (FB)	−0.15	**< 0.01**	2.29	−0.16	**< 0.01**	2.35
Psychological Barriers (PB)	−0.17	**< 0.01**	2.58	−0.18	**< 0.01**	2.62
ENV x FB				0.06	**< 0.01**	2.55
ENV x PB				0.01	0.78	2.46
MENF-AP x FB				0.06	**< 0.01**	3.00
MENF-AP x PB				0.01	0.61	2.91
MENF-AV x FB				0.09	**< 0.01**	3.39
MENF-AV x PB				0.03	0.21	3.36
HTAS x FB				−0.02	0.13	2.26
HTAS x PB				0.02	0.16	2.20
Model Diagnostics	ΔF_(18, 4783)_ = 206.97, **p-val < 0.01** R^2^ = 0.44 Adjusted R^2^ = 0.44, SE = 0.65			F_(26,4775)_ = 153.31, **p-val < 0.01** ΔF_(8, 4775)_ = 18.75, **p-val < 0.01** R^2^ = 0.45 Adjusted R^2^ = 0.45, SE = 0.64 ΔR^2^ = 0.02, **p-val < 0.01**		

Note. ^a^Reference Category: Female; ^b^Reference Category: Spain; ^c^Reference Category: Employed; ^d^Reference Category: Below Post-Secondary; ^e^Reference: Urban; Significant values marked in bold

The regression model showed that age was somehow positively driving consumption intentions, with younger individuals less likely to have higher consumption intentions for NUS. Other socio-demographic characteristics showed negative associations, such as being male (compared to females) and residing in certain countries (compared to residing in Spain), thereby mirroring the country-level differences earlier registered (Table 2).

Upon examining the other variables, approach motivations towards new food seemed to be a positive predictor for consumption intentions, together with environmental beliefs and health and taste attitudes towards food. Conversely, functional and psychological barriers were negative drivers of consumption intentions. Incidentally, avoidance motives towards new food seemed to be positively linked to consumption intentions, although at a small effect.

When considering interaction effects in the hierarchical regression (Table 4), only functional barriers seemed to have initial evidence of moderating relationships between environmental beliefs, approach motivations, and avoidance motivations towards consumption intentions for NUS. The significant interaction terms in the model may signify that driving factors towards NUS consumption intentions are contingent on how strongly the participants perceived functional barriers. Put together, these findings provide partial support for H1-3 of this study.

### Demographic differences and trends in perceived barriers to consumption

Finally, three consumer segments emerged after running the cluster analysis procedure (Table 5). The first, “Reluctant Consumers,” showed high perceived barriers and low drivers. The second, “Mild Explorers,” had moderate perceptions. The third, “Eager Consumers,” reported the lowest perceived barriers and the strongest drivers for consuming NUS products.

**Table 5:** Cluster profiling and comparison results (*n* = 4802)

Variable	Cluster 1: “Reluctant Consumers” *N* = 2105 (43.8%)	Cluster 2: “Mild Explorers” *N* = 1328 (27.7%)	Cluster 3: “Eager Consumers” *N* = 1369 (28.5%)	*p*-val
**Cluster Variable Means**, $\mathbf{\bar X}$	HTAS = 3.0 ENV = 3.4 APP = 2.8 AVD = 2.7 FB = 3.1 PB = 3.1	HTAS = 3.2 ENV = 4.2 APP = 3.5 AVD = 1.8 FB = 2.8 PB = 2.7	HTAS = 3.5 ENV = 4.3 APP = 4.3 AVD = 1.5 FB = 2.2 PB = 2.0	All *p* < 0.01
**Cluster Profiles**				
**NUS Intentions**	3.1	3.5	4.1	**< 0.01** η² = 0.23
**Age**,** years**	43.6	43.3	42.3	**0.012** η² = 0.002
**Gender** Male Female	1171 (55.6%) 932 (44.3%)	601 (45.3%) 727 (54.7%)	602 (44.0%) 767 (56.0%)	**< 0.01** χ2_(4)_ = 61.0
**Education Level** Up to Primary Education Secondary Education Post-secondary, non-tertiary education University Degree	85 (4.0%) 1236 (58.7%) 368 (17.5%) 416 (19.8%)	30 (2.3%) 752 (56.6%) 275 (20.7%) 271 (20.4%)	22 (1.6%) 705 (51.5%) 279 (20.4%) 363 (26.5%)	**< 0.01** χ2_(10)_ = 54.0
**Occupation** Unemployed Student Self-Employed Part-Time Full-Time Retired Other	241 (11.4%) 129 (6.1%) 129 (6.1%) 199 (9.5%) 1094 (52.0%) 205 (9.7%) 108 (5.1%)	133 (10.0%) 124 (9.3%) 79 (5.9%) 137 (10.3%) 665 (50.1%) 116 (8.7%) 74 (5.6%)	117 (8.5%) 131 (9.6%) 106 (7.7%) 129 (9.4%) 729 (53.3%) 109 (8.0%) 48 (3.5%)	**< 0.01** χ2_(12)_ = 39.6
**Area** Rural Urban	749 (35.6%) 1356 (64.4%)	421 (31.7%) 907 (68.3%)	409 (29.9%) 960 (70.1%)	**< 0.01** χ2_(2)_ = 13.4

From Table 5, there seem to be associations specific to each cluster. “Reluctant Consumers” included relatively more males compared to “Eager Consumers” with more females. Age and residence area were also similarly distributed across clusters. At a country level, “Eager Consumers” were more concentrated in countries such as Austria whereas the “Reluctant Consumers” were mostly based in the Czech Republic. These findings altogether provide support for H4 of this study (Table 6).

**Table 6:** Distribution of (a) *Reluctant*, (b) *Mild*, and (c) *Eager* respondents in EU countries, in % (*n* = 4802)

Country	(a) Reluctant Consumers	(b) Mild Consumers	(c) Eager Consumers
Austria	14.8	14.7	21.4
Belgium	17.3	14.1	15.0
Italy	15.1	14.8	17.0
Serbia	16.0	15.0	18.1
Spain	15.3	23.0	15.7
Czech Republic	21.5	18.4	12.8

Note. Columns sum up to 100%

## Discussion

The evidence in this study suggests that psychological and functional barriers significantly influenced an individual’s NUS consumption intentions in a comparable degree, similar to prior studies^43,44^. Consistent with the Innovation Resistance Theory, this study demonstrates the joint influence of both barriers towards NUS. Beyond mapping these theoretical barriers, this study also captured geographical differences on consumers’ motivations to eat other food products, similar to previously recorded literature on food choice and avoidance behaviors^42,45^. Consequently, these may possibly stem from various norms in eating, as well as with the regulation of food consumption by institutions in each context^46,47^.

Another key finding from this study was that approach motivations to new food and environmental beliefs were significant positive drivers of NUS consumption intentions. Evidently, there is emerging interest among consumers for food products linked to sustainability, and sustainable consumption motives seem to be driven by growing concerns for the environmental impact of food products^48^. However, some evidence in the literature still point out that consumers are unaware of the environmental impacts of food products^49^. Therefore, for NUS, sensitizing consumers to their environmental benefits might enhance their prominence.

This study also showed how health and taste preferences can strongly influence the consumption of NUS, similar to other foods like organic products^50^. Hence, the inherent health properties of NUS can be further emphasized, especially since some consumers perceived safety and nutritional issues for NUS^7,19,51^. Furthermore, leveraging the competitive dietary advantages of NUS in comparison to conventional products may be quite effective^52,53^.

Beyond these relationships, initial evidence of interaction effects was seen with functional barriers regarding environmental concerns and motivations to eat new food in particular. This points out that the functional use of NUS needs to be further clarified among consumers when positioning these products as new or beneficial to the environment. Moreover, the functional barriers operationalized in this study dealt with unknowing and concern, which may be indirectly linked to an individual’s curiosity about these products. An individual’s heightened curiosity towards food products has been observed to drive behaviors despite risks, as well as to cause further indulgence and willingness to consume products^54–56^. Despite these findings, it is also critical to consider supply chain aspects of NUS. While there may be different recorded functional or psychological barriers for NUS, ensuring market access and availability is a crucial aspect that can potentially involve more consumers to different food^57,58^. Being unreachable in the market may render consumers unaware of these products, and consequently, they may not be conscientious of the potential benefits associated with the consumption of NUS^59^. In this study, the focus was scoping barriers that consumers perceive when considering primarily the product, but nevertheless, NUS supply chains need to be reinforced so that they may be made more apparent among consumers in their routine food choice decision-making especially since NUS products already suffer from the lack of market visibility to date.

Interestingly, avoidance motivations for new food were seen in this study as positive drivers of consumption intentions to NUS. This contrasts with prior studies which have demonstrated that avoidance motivations can deter consumption. For example, the reluctance and aversion towards new and emerging products food experiences has been observed to restrict the positive perceptions towards food consumption, alongside notions of unfamiliarity or even disgust^32,60^. While the data in this study revealed somewhat otherwise, it demonstrates the dynamics of approach and avoidance that can result in consumer interest due to uncertainties^34^.

In the analysis of consumer clusters, the “Eager Consumers” group for products with NUS was typically composed more of females. A similar profile was also observed for groups of individuals that were more or less inclined to consume plant-based food products, or even those inclined to eat organic products in prior literature^61,62^. Furthermore, semblances of cross-national differences in the consumption of pulses were also found in a prior study^63^. This study further argues that it is important to devise an encompassing approach in communicating the benefits of these food products, even initially in specific communities where “Eager Consumers” reside.

While this study explored further the potential of NUS, there are some limitations that must be considered. This study relied on an online consumer survey which captured self-reported measures only, and as such, this poses risks on desirability biases among the respondents^64^. Multiple samples in different countries also require careful consideration of prevailing social contexts to understand differences in demographic profiles. Moreover, the large sample size in this study warrants some consideration. With larger samples, the extent of significant differences may be diminished and attributed mostly to minute differences rather than substantial variations. To this end, the interpretation of the statistical findings was compounded with reported effects sizes to better position the findings. From the results discussed, small and medium effect sizes were noted, yet the outcomes generally revealed what kinds of factors can affect consumption intentions for NUS. These findings then can be used as a springboard for practical guidelines in mitigating barriers to NUS consumption while activating motivators simultaneously. Nevertheless, the sampling procedure done in this study established representativity for the six countries studied for age and gender. The large sample size also allowed for increased likelihoods of obtaining true effects, thereby leading to further generalizability. Other means of avoiding common method biases were put in place such as pre-testing the survey, using validated scales, reverse-scoring, and controlling for response quality through monitoring global survey response times.

Some future research directions can be emphasized. Future studies can identify a more precise typology of NUS as this may elicit complementary or antagonistic perceptions from consumers. This will complement existing work done on other crop varieties as some studies have also explored drivers and barriers to other food products such as pulses^63^. Finally, more precise clustering studies can be forwarded to identify markets which are highly probable of easily incorporating NUS into the diet.

To conclude, this study identified different types of barriers towards NUS consumption alongside driving factors that may increase the prominence of NUS products to a broader audience. On a practical note, this study suggests synergistic interventions that aim to reduce psychological and functional barriers while also activating personal motivations to consume NUS. Based on the study’s findings, example interventions can be marketing strategies that demonstrate the nutritional potential of NUS preparations which also highlight their competitive advantages as compared to conventional crops. In another example, since this study showed that some consumers perceive conventional products to be enough, communication can also be made regarding the diverse uses of NUS for familiar applications. Furthermore, while consumers may not have had strong negative scores in this study regarding their intentions to consume NUS, this study showed that these products can be positioned based on their health and environmental benefits to maximize appeal. Moreover, this study also illustrated possible promotional strategies that are contingent on consumer preferences. For reluctant consumers, for instance, focusing on lowering perceived barriers may be beneficial, whereas for more eager consumers towards NUS, promoting the environmental benefits linked with NUS may be more strategic than considering them as a “new” food. Ultimately, aligning consumer preferences with the many benefits associated with consuming NUS can improve their salience.

## Methods

### Participants and procedures

Cross-sectional survey data were collected online across six different European countries that cover different geographical zones in the region (Austria, Belgium, Czech Republic, Italy, Serbia, Spain). In sampling participants, quotas were set for age groups and gender. The survey also only targeted individuals who were ‘mainly’ or ‘partially responsible’ for grocery shopping in the household and older than 18 years. Ethical clearance was received prior to initiating the survey process, as granted by the Czech University of Life Sciences Prague Ethics Committee (Ref. No. 022023), and all methods were carried out according to the prevailing guidelines and regulations, as well as with the Declaration of Helsinki. Further, informed consent was requested from all participants in this study.

To aid in the data collection procedure, Talk Online Panel s.r.o., a market research agency compliant with ESOMAR statutes and GDPR principles, was involved in translating and disseminating the survey to consumer panels. Incomplete responses, too rapid or slow responders, and missing data were removed from the subsequent analyses. Eventually, a total number of 4802 respondents were able to successfully complete the survey, and the respondents’ characteristics are in Table 7.

**Table 7:** Socio-demographic characteristics of overall study sample (*N* = 4802)

Characteristics		Austria *N* = 800	Belgium *N* = 757	Czech Rep. *N* = 824	Italy *N* = 750	Serbia *N* = 889	Spain *N* = 782
Age, years	X̄ ± S.D.	44.1 ± 13.4	43.1 ± 13.5	43.8 ± 12.4	43.3 ± 13.1	42.9 ± 13.1	41.9 ± 12.4
Household Size, total	X̄ ± S.D.	2.5 ± 1.3	2.6 ± 1.3	2.8 ± 1.2	3.0 ± 1.1	3.3 ± 1.4	3.1 ± 1.1
Gender (%)	Male	400 (50)	373 (49.3)	414 (50.2)	369 (49.2)	433 (48.7)	385 (49.2)
	Female	400 (50)	384 (50.7)	408 (49.5)	381 (50.8)	456 (51.3)	397 (50.8)
Education Level (%)	Up to Primary Education	9 (1.1)	28 (3.7)	28 (3.4)	32 (4.2)	15 (1.7)	24 (3.0)
	Up to Secondary Education	507 (63.4)	371 (49.0)	569 (69.1)	432 (57.6)	570 (64.1)	244 (31.2)
	Post-Secondary, Non-Tertiary	157 (19.8)	153 (20.2)	78 (9.5)	156 (20.8)	94 (10.6)	283 (36.2)
	University Degree	126 (15.8)	204 (26.9)	149 (18.1)	130 (17.3)	210 (23.6)	231 (29.5)
	No Formal Education	0	1 (0.1)	0	0	0	0
Residence Area (%)	Rural	399 (49.9)	344 (45.4)	336 (40.8)	228 (30.4)	138 (15.5)	134 (17.1)
	Urban	401 (50.1)	413 (54.6)	488 (59.2)	522 (69.6)	751 (84.5)	648 (82.9)
Occupation (%)	Unemployed	23 (2.9)	88 (11.6)	24 (2.9)	121 (16.1)	109 (12.3)	126 (16.1)
	Student	63 (7.9)	71 (9.4)	44 (5.3)	74 (9.9)	76 (8.5)	56 (7.2)
	Self-Employed	43 (5.4)	31 (4.1)	66 (8.0)	79 (10.5)	56 (6.3)	39 (5.0)
	Part-time Employee	127 (15.9)	85 (11.2)	53 (6.4)	87 (11.6)	22 (2.5)	91 (11.6)
	Full-time employee	385 (48.1)	340 (44.9)	507 (61.5)	305 (40.7)	547 (61.5)	404 (51.7)
	Retired	125 (15.6)	76 (10.0)	90 (10.9)	35 (4.7)	65 (7.3)	39 (5.0)
	Other	34 (4.3)	66 (8.7)	40 (4.9)	49 (6.5)	14 (1.6)	27 (3.5)

### Measures

In the consumer survey, respondents were first introduced to the concept of neglected and underutilized species, focusing on their relatively unknown status in comparison to conventional crops. Afterwards, the consumers proceeded to answering the different scales related to the topic of NUS. From the conceptual framework (Fig. 1), barriers to innovation (in the case of NUS products), both functional (FB) and psychological (PB) in nature, were measured through a 5-point Likert scale (1: Strongly Disagree – 5: Strongly Agree). Statements referring to these barriers were based on studies that dealt with food consumption barriers, but adapted to the context of NUS^4,28,29,42,63^.

The other psychological variables in this study were represented as ‘MENF-AP’, ‘MENF-AV’, ‘HTAS’, and ‘ENV’. The first two were approach and avoidance motivations to eat new food, derived from the validated MENF questionnaire^34^. Next, the Health and Taste Attitudes (HTAS) questionnaire was used, particularly on the statements pertaining to the general health and natural product interests of consumers towards food^35^. Finally, environmental beliefs (ENV) were assessed with statements adapted from similar studies^65,66^. These variables were also measured using a 5-point Likert scale (1: Strongly disagree – 5: Strongly agree).

The outcome variable of interest in this study is the intention to consume NUS products of the consumers, ‘INT’. This was also measured through a 5-point Likert scale (1: Strongly disagree – 5: Strongly agree), and was operationalized into statements that aimed to capture the overall intention levels of individuals to eat more of these products. The relevant measures used in the survey are compiled in Appendix A2 of this article.

### Data analysis

Country level comparisons among the sample regarding the perceived barriers and drivers to consumption as well as consumption intentions of NUS products were first identified and subjected to difference testing through ANOVA procedures with post-hoc tests (Games-Howell), considering the resulting variance homogeneity and different sample sizes. Afterwards, exploratory factor analyses were done to estimate how the survey statements on consumption barriers loaded on the proposed conceptual barrier type. In this case, functional and psychological barriers were treated as distinct and separate constructs based on the Innovation Resistance Theory. Separating the factor analyses was done to preserve these conceptual structures from theory, and also to minimize potential artificial cross-loadings that could arise for the barrier types. The extraction of factors was based on principal components and a varimax rotation was selected. Furthermore, factor extraction was done considering the reported eigenvalues (> 1; Kaiser criterion), in line with the elbow in the scree plots. Next, summated scales were constructed for the variables of interest to have a composite measure for each and scale reliability measures were also obtained. Pearson’s correlation coefficients were then assessed to check for significant associations among the variables in the study.

Hierarchical multiple regression models were then built with consumption intentions for NUS as the dependent variable (INT) and barriers and drivers to consumption as the independent variables (PB, FB, MENF-AP, MENF-AV, HTAS, ENV) together with socio-demographic characteristics. In the first block, all independent variables were mean-centered and entered in the model to determine linear additive effects towards the dependent variable^67^. Interaction effects across the perceived barriers and drivers were included consecutively to assess potential moderating influences. Diagnostics (Adj. R^2^), collinearity statistics (VIF), and residuals were calculated to assess model accuracy^67^.

Finally, a hierarchical cluster analysis with Ward’s method using the squared Euclidean distances was performed based on the respondents’ perceived psychological and functional barriers to consuming NUS products, together with the identified drivers discussed previously^68^. For all the statistical procedures performed, SPSS (V.29.0) was used, and a critical alpha level of 5% was defined for all relevant tests.

## Supplementary Information

Below is the link to the electronic supplementary material.

Supplementary Material 1

## Data Availability

The data that support the findings of this study are produced under the H2020 Cropdiva Project, but restrictions apply to the availability of these data, and so are not publicly available. The data are, however, may be available from the authors upon reasonable request and with the permission of the H2020 Cropdiva consortium.
