# Supplementary material for: Barriers and drivers to consuming neglected and underutilized species: evidence from six European countries
Source: Sci Rep. 2025 Nov 19;15:40698. doi: 10.1038/s41598-025-24443-4 (PMC12630740; doi:10.1038/s41598-025-24443-4)
Supplement: Supplementary file 1 — Supplementary Material 1 [file 41598_2025_24443_MOESM1_ESM.docx]

## **Barriers and Drivers to Consuming Neglected and Underutilized Species: Evidence from six European countries**

## **APPENDIX**

**A1. Comparison of (a) functional and (b) psychological barriers to NUS consumption**


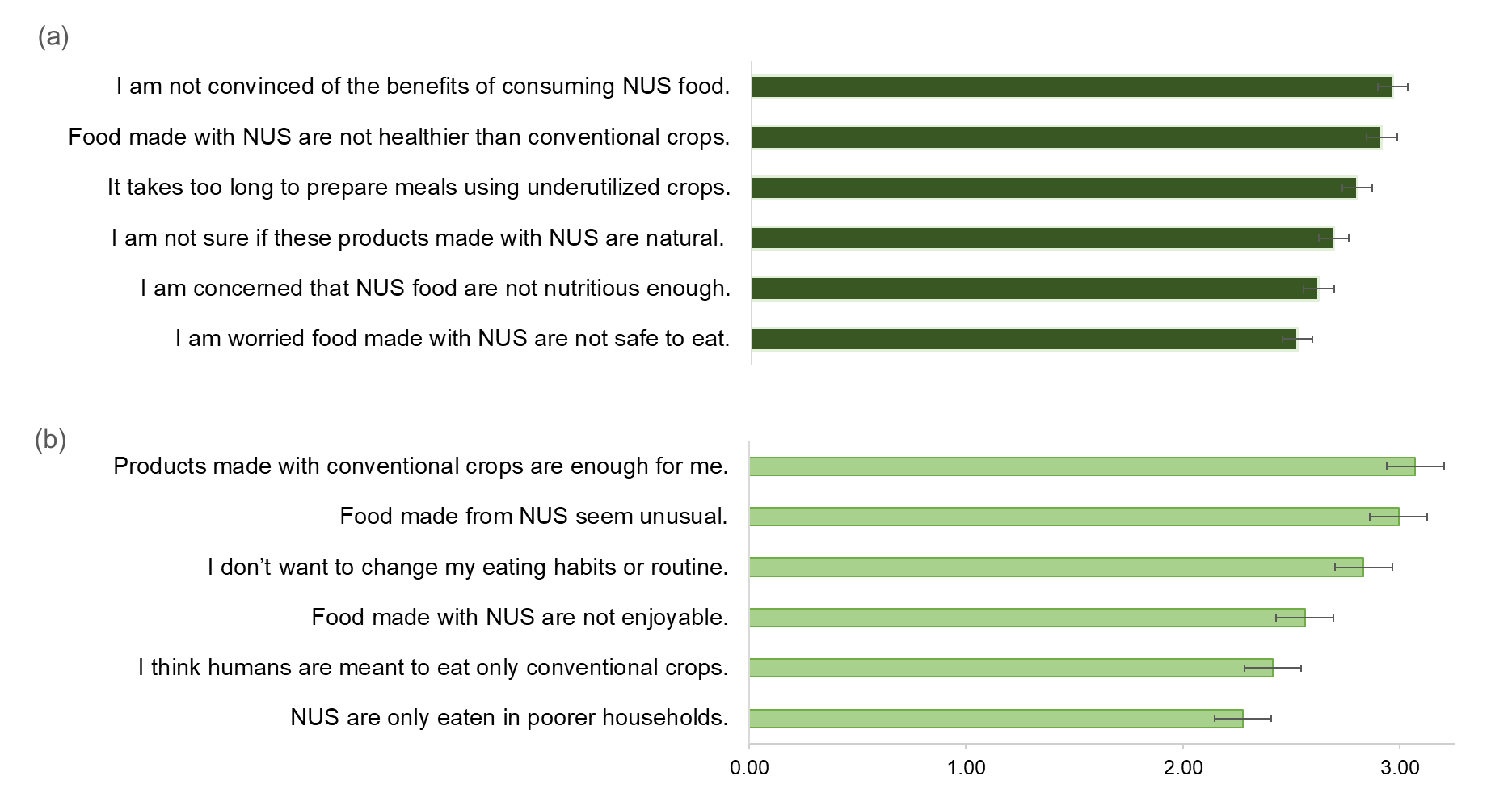


Note. Values are presented as Means ± SD. The extent of barriers perceived were measured through a 5-point Likert scale (1: Strongly disagree to 5: Strongly agree).

**A2. Scales for Measures Used**

All statements were evaluated by the survey respondents through 5-point Likert scales.

*“To what extent do you agree with the following statements?”*

| **Variables** | **Label** | **Survey Statements** | **Sources** |
| --- | --- | --- | --- |
| FB | FB1 | I am worried food made with underutilized crops are not safe to eat. | 1,2,2–4 |
|  | FB2 | I am concerned that underutilized crops are not nutritious enough. |  |
|  | FB3 | I am not sure if these products made with underutilized crops are natural. |  |
|  | FB4 | I am not convinced of the benefits of consuming food made with underutilized crops. |  |
|  | FB5 | It takes too long to prepare meals using underutilized crops. |  |
|  | FB6 | Food made with underutilized crops are not healthier than conventional crops. |  |
| PB | PB1 | I think humans are meant to eat only conventional crops. |  |
|  | PB2 | I don’t want to change my eating habits or routine. |  |
|  | PB3 | Products made with conventional crops are enough for me. |  |
|  | PB4 | Food made with underutilized crops are not enjoyable. |  |
|  | PB5 | Underutilized crops are only eaten in poorer households. |  |
|  | PB6 | Food made from underutilized crops seem unusual. |  |
| INT | INT1 | I intend to try products made with underutilized crops. |  |
|  | INT2 | I want to incorporate more products made with underutilized crops in my diet. |  |
|  | INT3 | I expect to regularly eat products made with underutilized crops. |  |
| MENF-AP | MAP1 | I enjoy trying foods that I have never eaten before. | 5 |
|  | MAP2 | I enjoy learning about new foods |  |
|  | MAP3 | I am interested in trying familiar foods that have been prepared with new ingredients. |  |
|  | MAP4 | I am curious about the flavors of new foods and ingredients. |  |
|  | MAP5 | I get sort of excited when I know I am going to eat some new types of food. |  |
| MENF-AV | MAV1 | I don’t trust new foods |  |
|  | MAV2 | I am afraid to eat things I have never had before |  |
|  | MAV3 | I think that if I eat something I have not eaten before that it will taste strange. |  |
|  | MAV4 | I think that if I eat something I have not eaten before that I will not like it. |  |
|  | MAV5 | Foods I have never eaten before seem sort of disgusting. |  |
| HTAS | HT1 | The healthiness of food has little impact on my food choices. | 6 |
|  | HT2 | I am very particular about the healthiness of food I eat. |  |
|  | HT3 | I eat what I like and I do not worry much about the healthiness of food. (R) |  |
|  | HT4 | It is important for me that my diet is low in fat. |  |
|  | HT5 | I try to eat foods that do not contain additives. |  |
|  | HT6 | I do not care about additives in my daily diet. (R) |  |
|  | HT7 | I do not eat processed foods, because I do not know what they contain. |  |
| ENV | ENV1 | I am convinced that global warming is happening. | 7,8 |
|  | ENV2 | I personally worry about the effects of global warming. |  |
|  | ENV3 | It is better for the environment if we regulate our food consumption. |  |

(R) indicates that the item was reverse coded and corrected in the scoring procedure.

Sources Cited

1. Giacalone, D., Clausen, M. P. & Jaeger, S. R. Understanding barriers to consumption of plant-based foods and beverages: insights from sensory and consumer science. *Current Opinion in Food Science* **48**, 100919 (2022).

2. Kaur, P., Dhir, A., Ray, A., Bala, P. K. & Khalil, A. Innovation resistance theory perspective on the use of food delivery applications. *Journal of Enterprise Information Management* **34**, 1746–1768 (2020).

3. Kushwah, S., Dhir, A. & Sagar, M. Understanding consumer resistance to the consumption of organic food. A study of ethical consumption, purchasing, and choice behaviour. *Food Quality and Preference* **77**, 1–14 (2019).

4. Perez-Cueto, F. J. A. *et al.* How barriers towards plant-based food consumption differ according to dietary lifestyle: Findings from a consumer survey in 10 EU countries. *International Journal of Gastronomy and Food Science* **29**, 100587 (2022).

5. Nezlek, J. B., Forestell, C. A. & Cypryanska, M. Approach and avoidance motivation and interest in new foods: Introducing a measure of the motivation to eat new foods. *Food Quality and Preference* **88**, 104111 (2021).

6. Roininen, K., Lähteenmäki, L. & Tuorila, H. Quantification of consumer attitudes to health and hedonic characteristics of foods. *Appetite* **33**, 71–88 (1999).

7. Leiserowitz, A., Shome, D., Marx, S., Hammer, S. & Broad, K. *New York City Global Warming Survey*. https://climatecommunication.yale.edu/publications/new-york-city-global-warming-survey/ (2008).

8. Spendrup, S. & Hovmalm, H. P. Consumer attitudes and beliefs towards plant-based food in different degrees of processing – The case of Sweden. *Food Quality and Preference* **102**, 104673 (2022).
